# Deep learning–enabled fluorescence imaging for surgical guidance: *in silico* training for oral cancer depth quantification

**DOI:** 10.1117/1.JBO.30.S1.S13706

**Published:** 2024-09-18

**Authors:** Natalie J. Won, Mandolin Bartling, Josephine La Macchia, Stefanie Markevich, Scott Holtshousen, Arjun Jagota, Christina Negus, Esmat Najjar, Brian C. Wilson, Jonathan C. Irish, Michael J. Daly

**Affiliations:** a University Health Network, Princess Margaret Cancer Centre, Toronto, Ontario, Canada; b University of Toronto, Department of Otolaryngology-Head and Neck Surgery, Toronto, Ontario, Canada; c University of Toronto, Department of Medical Biophysics, Toronto, Ontario, Canada

**Keywords:** molecular-guided surgery, fluorescence imaging, spatial frequency domain imaging, optical tomography, deep learning, oral cancer surgery

## Abstract

**Significance:**

Oral cancer surgery requires accurate margin delineation to balance complete resection with post-operative functionality. Current *in vivo* fluorescence imaging systems provide two-dimensional margin assessment yet fail to quantify tumor depth prior to resection. Harnessing structured light in combination with deep learning (DL) may provide near real-time three-dimensional margin detection.

**Aim:**

A DL-enabled fluorescence spatial frequency domain imaging (SFDI) system trained with *in silico* tumor models was developed to quantify the depth of oral tumors.

**Approach:**

A convolutional neural network was designed to produce tumor depth and concentration maps from SFDI images. Three *in silico* representations of oral cancer lesions were developed to train the DL architecture: cylinders, spherical harmonics, and composite spherical harmonics (CSHs). Each model was validated with *in silico* SFDI images of patient-derived tongue tumors, and the CSH model was further validated with optical phantoms.

**Results:**

The performance of the CSH model was superior when presented with patient-derived tumors (P-value<0.05). The CSH model could predict depth and concentration within 0.4 mm and 0.4  μg/mL, respectively, for *in silico* tumors with depths less than 10 mm.

**Conclusions:**

A DL-enabled SFDI system trained with *in silico* CSH demonstrates promise in defining the deep margins of oral tumors.

## 1 Introduction

Of the 10 most common solid tumors, oral cancer has the highest prevalence of positive margins following surgery.^1^ This has a negative impact on patient outcomes, as clear surgical margins are a consistent prognostic factor for local control and overall survival in oral cancer.^2^^,^^3^ Surgical intervention is required for most oral cancer patients, yet tumor delineation remains a significant challenge.^4^^,^^5^ With these procedures, a balance is required between complete tumor resection and conservation of healthy tissue to prevent tumor recurrence and preserve functionality.^6^ Although palpation, visual cues, pathology, and anatomical imaging modalities are common intraoperative techniques to define the tumor, these methods are limited in their precision; consequently, the incidence of positive margins in oral cancer has been reported as high as 85%.^7^^,^^8^

Fluorescence-guided surgery is emerging as an intraoperative imaging technique for cancer surgery, as it provides high-resolution contrast to differentiate tumor from healthy tissue.^9^ A growing number of fluorescence contrast agents are approved or currently under investigation across a wide spectrum of surgical specialities.^10^ In oral cancer, recent clinical trials are establishing fluorescence imaging for intraoperative assessment of surgical margins.^11^^,^^12^ The use of near-infrared (NIR) fluorophores (650 to 900 nm) enables visualization of sub-surface structures due to lower absorption in the biological imaging window.^13^ Although this can provide surgical guidance in many clinical scenarios, one key limitation is that clinical fluorescence devices do not quantify the depth of sub-surface structures.^14^^,^^15^ This is particularly important for oral cancer, as determining the depth of tumor invasion is often the key challenge leading to positive margins.^16^ The sentinel margin technique developed by van Keulen et al. is an important advance to help identify the closest sub-surface margin based on relative fluorescence measurements of the *ex vivo* surgical specimen,^17^^,^^18^ but as yet this lacks quantitative capabilities. Results from a phase II fluorescence guidance trial demonstrate the challenge of oral cancer depth assessment.^11^ Specifically, de Wit et al. report that the accuracy of margin classification degraded as margin thickness increased (i.e., from 1 to 3 mm to 3 to 5 mm of overlaying tissue in post-resection surgical specimens). These same authors also suggest that fluorescence depth detection might improve results, citing the potential for variable aperture (VA) and spatial frequency domain imaging (SFDI) techniques.^11^

Several imaging techniques are under development for intraoperative fluorescence depth detection. Using VA, the dual-aperture fluorescence ratio technique recently reported by Rounds et al.^19^ accurately classified close versus clear margins in *ex vivo* surgical specimens of an initial patient cohort. For depth quantification using SFDI, techniques have been developed to quantify the depth to sub-surface inclusions based on the fluorescence decay rate across spatial frequencies.^20^^,^^21^ Alternative techniques using hyperspectral acquisition (i.e., multi-excitation and/or multi-emission wavelength bands) and ratiometric techniques have also been validated in pre-clinical neurosurgery models of buried tumors.^22^^–^^24^ All of these examples focus on identifying the top surface of buried tumors lying below the tissue surface. In these cases, which we refer to as a “submarine” model, depth is defined as the thickness of healthy tissue covering a fluorescent inclusion. These techniques have been motivated by clinical applications including guidance of neurosurgical procedures that involve “piecemeal” resection of layers of tissue overlying a buried tumor^20^^–^^24^ or for imaging the basal surface of an *ex vivo* surgical specimen.^19^^,^^25^ As oral cancer tends to originate at the mucosal surface and infiltrate deeper into healthy tissue,^16^ here, we focus on identifying the bottom surface of infiltrative tumors, which we refer to as an “iceberg” model. In this case, depth is the thickness of the fluorescent tumor, as would be encountered during *in vivo* imaging of the mucosal surface in oral cancer.

For depth quantification, we are using SFDI acquisition in combination with deep learning (DL) reconstruction. SFDI captures wide-field reflectance images while projecting patterns of light to enable quantification of the optical scattering and absorption properties of the tissue.^26^^,^^27^ SFDI fluorescence images provide depth information: high-frequency patterns capture shallow information, and low-frequency patterns obtain deeper information.^28^ Many existing fluorescence depth quantification approaches use ratiometric or analytical methods with approximations to simplify the inverse problem, such as assumptions of semi-infinite mediums or fluorescence originating from point-source inclusions.^20^^,^^22^^–^^24^ DL techniques offer the potential to use learning-based techniques to relax the requirement for mathematical simplicity or complex numerical inversion.^29^^,^^30^ Furthermore, although time-intensive to train, DL methods rapidly generate inferences after image acquisition, which lends itself to intraoperative applications. Here, we adopt an *in silico* training approach that directly incorporates the physics of light propagation into the training process.^31^^,^^32^ The lack of SFDI images of patient tumors and the requirement for a comprehensive training dataset presented a need for synthetic images. Here, we investigate a custom “Siamese” convolutional neural network (CNN) for tumor delineation and corresponding *in silico* model for training. In this initial investigation, we employ 10 patient contours from pre-operative radiological imaging to generate optical testing data for simulations and phantom experiments.

## 2 Methods

### 2.1 Spatial Frequency Domain Imaging System

**Fig. 1:**
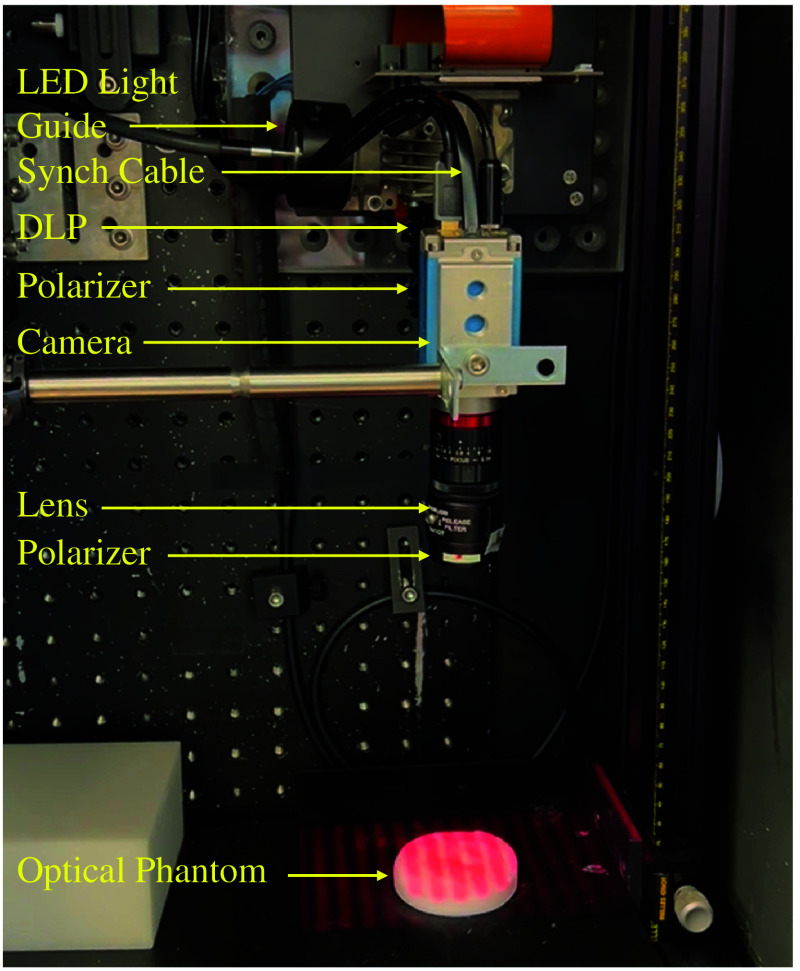
SFDI system prototype.

The prototype SFDI system under development is in Fig. 1. A light engine with six light-emitting diodes (LEDs) (Spectra X, Lumencor, Beaverton, Oregon, United States) that has 20-nm bandwidth sources centered at 390, 438, 475, 512, 586, and 632 nm is the light source for the system. A 3-mm liquid light guide (LGG0338, Thorlabs, Newton, New Jersey, United States) couples the light engine to the digital light projector (DLP), a spatial modulator (DLi6500 1080p Optics Bundle, DLi, Austin, Texas, United States) based on a 1920×1080-pixel DLP development kit (LightCrafter 6500, Texas Instruments, Dallas, Texas, United States). All images are captured with a 1392 × 1040-pixel, 14-bit, monochrome charge-coupled device (CCD) camera (Pixelfly USB, PCO AG, Kelheim, Germany) that is coupled to a 25-mm focal length lens (model 67715, Edmund Optics, Barrington, New Jersey, United States). A synchronization cable couples the projector and the camera so that images are acquired sequentially at each spatial frequency. In this study, the SFDI system collects fluorescence and reflectance images across six spatial frequencies $f_{x}=[0,0.05,0.1,0.15,0.2,\text{and }0.25]\;{\mathrm{mm}}^{-1}$ using the 632-nm light source. Reflectance images are required to obtain the optical properties of the tissue; absorption and scattering $\left(\mu_{a},\mu_{s}^{\prime }\right)$ are computed from reflectance images at two separate spatial frequencies $f_{x}=[0\text{ and }0.2]\;{\mathrm{mm}}^{-1}$ using SFDI lookup tables.^26^^,^^33^ A 650-nm-long pass filter (FELH0650, Thorlabs, Newton, New Jersey, United States) is used when capturing fluorescence images of protoporphyrin IX (PpIX). To remove specular reflection, the system includes crossed linear polarizers (LPVISE2X2, Thorlabs, Newton, New Jersey, United States). Prior to data acquisition, the SFDI system is calibrated with a polyurethane-based phantom containing titanium dioxide and carbon black (Biomimic PB0317, INO, Quebec City, Quebec, Canada).

### 2.2 Deep Learning Approach for Fluorescence Topography

**Fig. 2:**
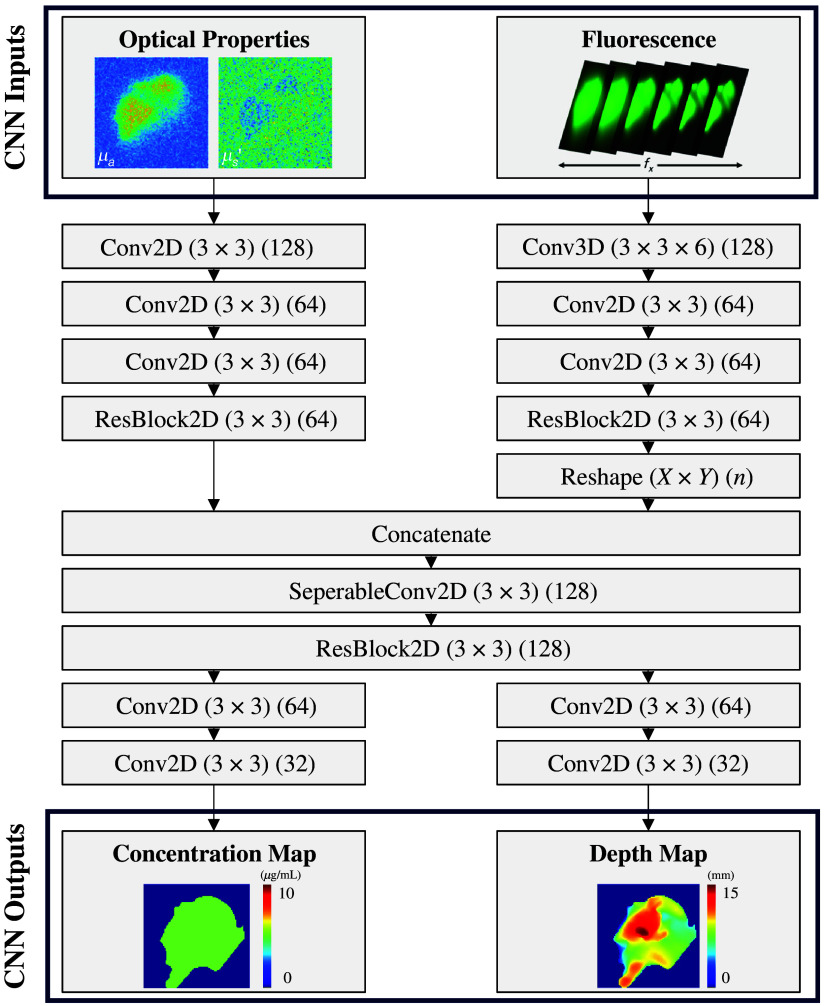
DL architecture and parameters. Depth and concentration maps are predicted from optical properties and fluorescence images collected from SFDI.

We investigate a DL model for obtaining fluorescence concentration and depth topography from SFDI images. The Siamese CNN architecture shown in Fig. 2 was adapted from the work done by Smith et al.^31^ in which DL was used to predict depth to the top surface of a submerged inclusion (“submarine” model) and the corresponding fluorescence lifetime from SFDI-derived optical properties and macroscopic fluorescence lifetime imaging. Our Siamese CNN differs as it uses optical properties and SFDI fluorescence images as separate inputs to predict the depth to the bottom surface of an infiltrative inclusion (“iceberg” model) and the corresponding fluorescence concentration. In addition to changing the model inputs and outputs, we also modified a few of the internal model details to improve performance, most notably changing 1×1 kernel sizes to 3×3 for improved deblurring and including residual blocks (ResBlocks) in both arms prior to concatenation.

As shown in Fig. 2, optical properties go through a series of two-dimensional (2D) convolutions with 3×3 filters, 1×1 stride, ReLU activation functions, and zero padding to maintain X×Y image dimensionality. Before concatenation, the images are fed into a 2D ResBlock in which two additional 2D convolutions are performed with a skip connection in which the input also bypasses the two sets of convolutions. Fluorescence images are passed to a single three-dimensional (3D) convolution with 3 × 3× 6 filters, 1×1×1 stride, a ReLU activation function, and zero padding to maintain dimensionality. These images are then passed to two 2D convolutional layers and a 2D ResBlock. As concentration and depth predictions depend on the optical properties and fluorescence images, the fluorescence data are reshaped and concatenated with the optical properties. A separable convolution and a 2D ResBlock are applied to the concatenated data. The concatenated data are split into two arms and a series of 2D convolutions that reduce the number of feature maps while maintaining X×Y image dimensionality and are applied to produce the fluorescence concentration map and depth map.

All DL implementation is in Amazon Web Services (AWS) SageMaker with a ml.g4dn.2xlarge instance type (1 NVIDIA T4 GPU, 8 vCPU). During training, 920,514 parameters are learned with Adam as the optimizer and learning rate decay (initial $\text{learning rate}=5\times 10^{-5}$). Training continued for a maximum of 100 epochs unless the early stopping criteria were met. Training the CNN took approximately 8.6 h at 311  s/epoch for the *in silico* datasets described below.

### 2.3 *In Silico* Training Data

**Fig. 3:**
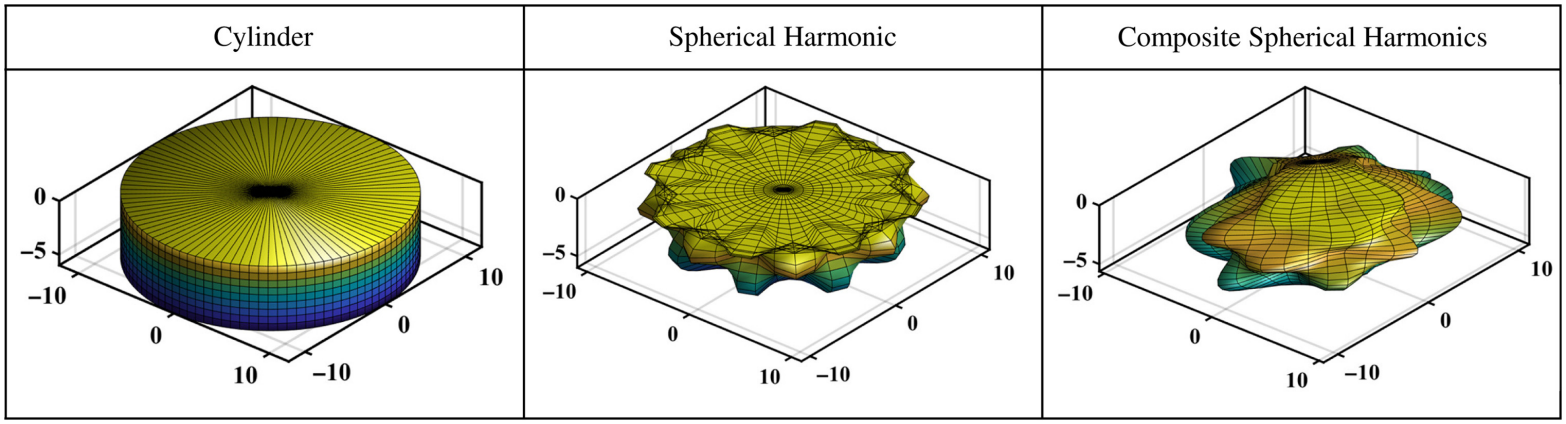
Shape models for deep learning training. All dimensions are in millimeters, and the color scale represents the relative maximum (blue) and minimum (yellow) depths of the inclusion.

**Fig. 4:**
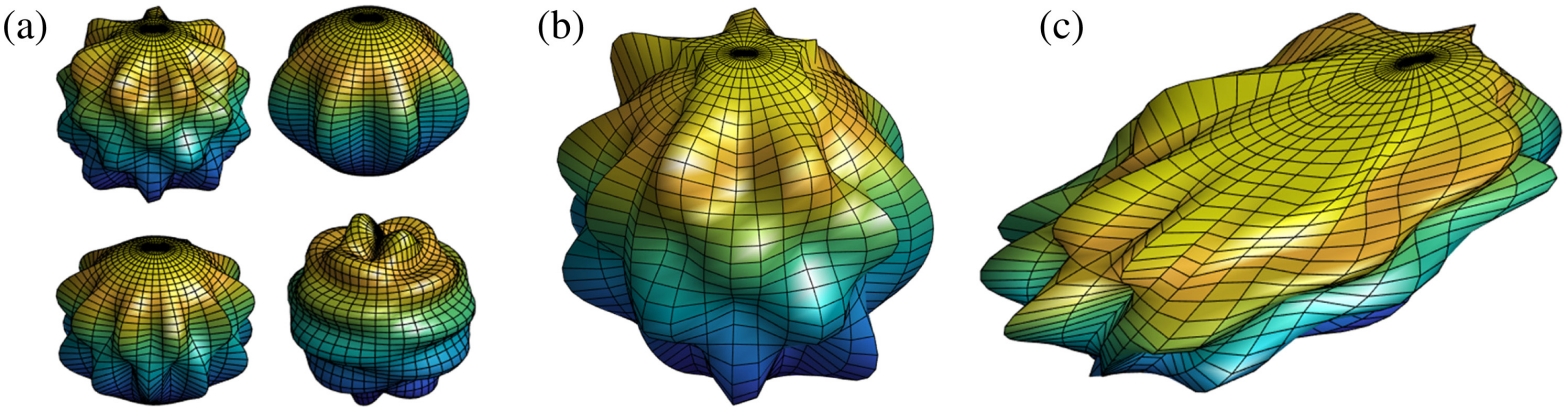
Method of generating a CSH. Four spherical harmonics are (a) randomly generated and (b) merged to create a base CSH, which is then (c) randomly shifted, scaled, rotated, and cut to create a final CSH. The color scale represents the relative maximum (blue) and minimum (yellow) depths of the shape at each step.

Figure 3 compares the three shape model iterations used to mimic oral cancer tumors: cylinders, spherical harmonics, and composite spherical harmonics (CSHs). A set of 10,000 of each shape model is generated in MATLAB. Cylinders were chosen as the first shape model as a proof of concept to assess the potential for DL in combination with fluorescence SFDI. Cylinders are generated with randomly chosen widths = 10 to 40 mm and depths = 1 to 10 mm, and to mimic the “iceberg” tumor model, the top surface of the cylinder is set to Z=0 (i.e., the tissue surface). Spherical harmonics were chosen as the second shape model as they could be quickly generated and introduced more complex geometric features. Spherical harmonics are originally generated with a radius of 10 mm and then scaled to have widths = 10 to 40 mm and depths = 1 to 10 mm. To create perturbations in the shape and diversity in the training set, the order and degree of the spherical harmonic are randomly selected with order = 2 to 20 and degree = order-20. To achieve the “iceberg” model, the section of the spherical harmonic protruding above Z=0 is cut. CSHs were developed to add asymmetry to the tumor model. The CSH model was intended to have organic features that varied significantly within the dataset. A CSH comprised four spherical harmonics joined as separate quadrants based on two randomly generated reciprocal functions in the XY plane as shown in Fig. 4. A series of transformations were included when developing the shape model to add regions of buried tumor for the primarily “iceberg” tumor. The CSH is rotated in the X, Y, and Z directions, shifted in the Z direction, scaled separately along X and Y axis to have widths = 10 to 40 mm, and scaled in the Z direction to have depths = 1 to 10 mm. The section of the CSH that is protruding over Z=0 is cut so that the tumor is flush with the background tissue.

All shape models were passed to an in-house tissue simulator based on a diffusion theory light propagation model in MATLAB to generate *in silico* images for DL training. Specifically, the simulator was based on a perturbation approach to model the tumor as a single-region heterogeneity using steady-state Green’s functions calculated on a 3D Cartesian grid.^34^^,^^35^ The tissue simulator produced *in silico* images over a field of view of $50\;{\mathrm{mm}}^{2}$ (0.5  mm/pixel resolution) with Poisson additive noise. A set of reflectance and fluorescence images across spatial frequencies, optical property maps, and true depth and concentration maps were generated for each synthetic tumor shape. The fluorescence and reflectance images were generated for six spatial frequencies $f_{x}=[0,0.05,0.1,0.15,0.2,\text{and }0.25]\;{\mathrm{mm}}^{-1}$ using PpIX properties for the fluorophore and a range of optical properties within tissue: absorption $\mu_{a}=0.0015$ to $0.015\;{\mathrm{mm}}^{-1}$ and scattering $\mu_{s}^{\prime }=0.75$ to $2\;{\mathrm{mm}}^{-1}$. Optical property maps were calculated using SFDI lookup tables with *in silico* reflectance images at $f_{x}=[0\text{ and }0.2]\;{\mathrm{mm}}^{-1}$. Fluorophore concentration was considered homogeneous and randomly chosen between 1 and 10  μg/mL, with an assumed PpIX quantum efficiency (η=0.046).^36^ Depth was calculated at each pixel; for areas in which the top surface of the tumor was at/above the healthy tissue (Z≥0), depth was calculated as the distance between the top surface and the bottom surface; for regions where the top surface of the tumor was buried (Z<0), depth was the distance from Z=0 to the bottom surface of the tumor. The cylinder, spherical harmonic, and CSH models used during *in silico* testing were trained with fluorescence solely in the tumor. A fourth training set based on CSH was developed for the phantom study and included a randomized amount of fluorescence in the healthy background tissue ranging from 0.1% to 50% of the fluorescence concentration of the tumor. Although the phantoms did not contain fluorescence in the background, this model provided better agreement with the experimental data that are subject to fluorescence filter leakage, camera electronic noise, and SFDI calibration errors not currently included in the training process. These factors led to a mismatch in background fluorescence between experimental and *in silico* SFDI images.

### 2.4 *In Silico* Implementation

To assess the feasibility of DL for predicting depth and concentration from SFDI fluorescence images and optical properties, *in silico* test sets were developed. A test set of 100 shapes was generated for each respective model (cylinder, spherical harmonic, and CSH). The geometric and optical properties remained within the range of the training data for all three sets. Images were passed to each model to confirm that the networks could make accurate predictions for new cases of the same data type as the training set.

**Fig. 5:**
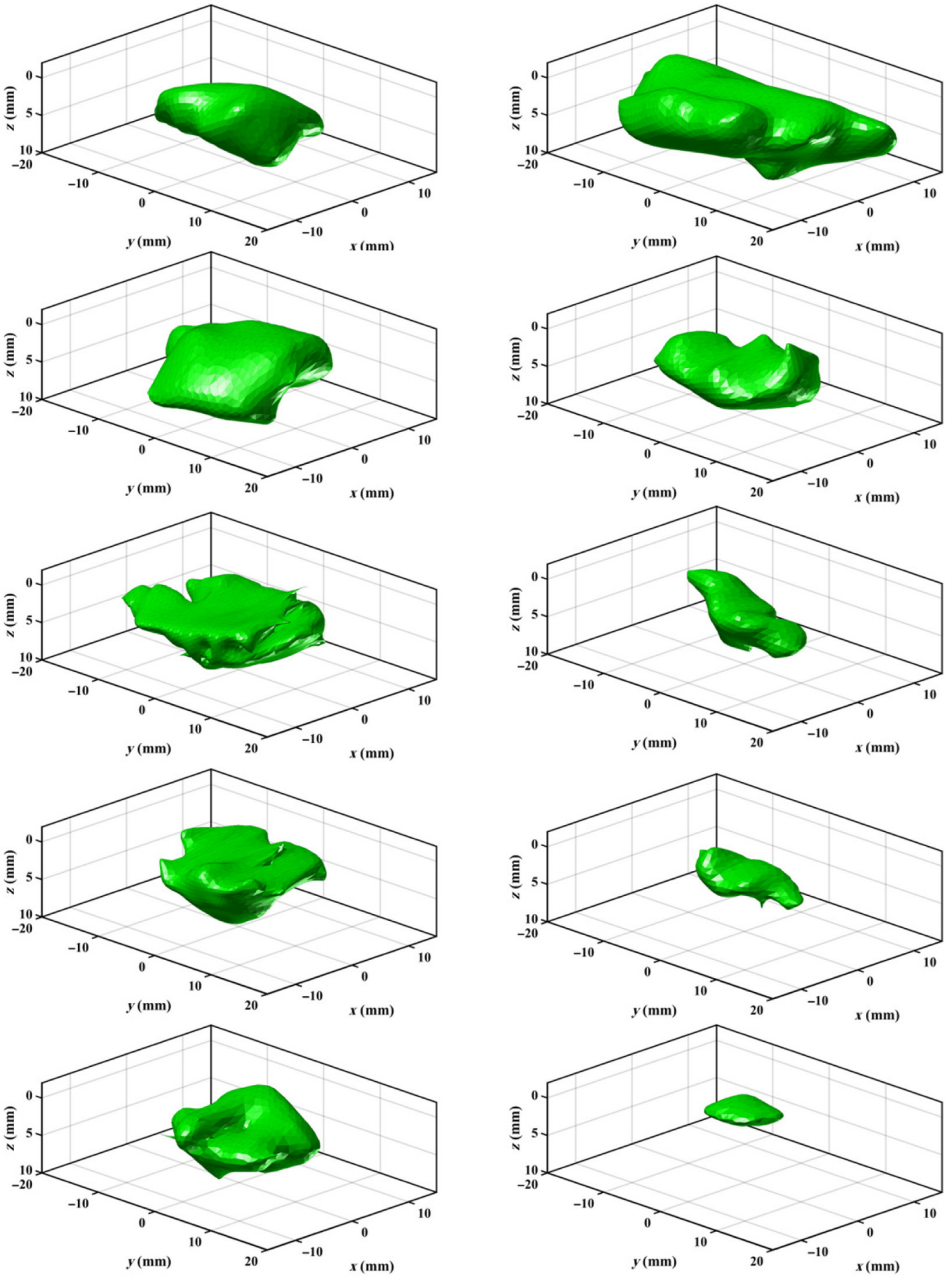
Validation set included 10 meshes of MRI-contoured patient tongue tumors.

Meshes of patient-derived oral cancer were used to evaluate the ability of the DL training data to capture complex features seen in real tumors. After institutional ethics board approval for retrospective patient data access (University Health Network REB #22-5471), 10 pre-operative magnetic resonance imaging (MRI) of tongue cancer surgical patients were contoured to produce corresponding meshes (average depth = 8.3 mm) as displayed in Fig. 5. Tongue cancer was selected as an initial model for evaluation as these are the most prevalent oral cancer subsite.^37^ The contoured tumors had widths and depths that fell within the geometric range of the training data. To increase the number of test samples, the 10 contours were scaled in each plane (widths = 10 to 40 mm and depths = 1 to 10 mm) and randomly rotated around the z axis (−180 to 180 deg). These transformations provided a second validation set of 100 patient-derived tumors (average depth = 5.4 mm). As the invasion depth of late-stage oral cavity cancer (e.g., T3–T4 tumors exceeding 1 cm depth) can exceed the penetration depths of near-infrared light, a third evaluation case was included based on an additional patient case (depth = 12.5 mm) to assess DL performance in out-of-range cases. The tumor exceeded the functional depth of our system but had widths that remained within the imaging field of view.

Both test sets were passed to the tissue simulator twice to produce a test set without background fluorescence and a test set with a randomized amount of fluorescence in the background (0.1% to 50% of the fluorescence concentration of the tumor). The sets with background fluorescence were used to assess the *in silico* performance of the model trained with CSH and background fluorescence. The tissue simulator produced synthetic fluorescence images (PpIX concentration=5  μg/mL), reflectance images, optical properties ($\mu_{a}=0.0045\;{\mathrm{mm}}^{-1}$, $\mu_{s}^{\prime }=1\;{\mathrm{mm}}^{-1}$), concentration maps, and depth maps for each test set. The patient tumors protruded above the z=0 plane, the depth for each pixel was calculated as the difference between the bottom surface of the tumor, and the top surface, which was either the Z=0 plane or the top surface of the protruding tumor. Images were passed to each DL model to assess how well the training data could capture the complex geometry of real patient tumors.

### 2.5 Phantom Implementation

**Fig. 6:**
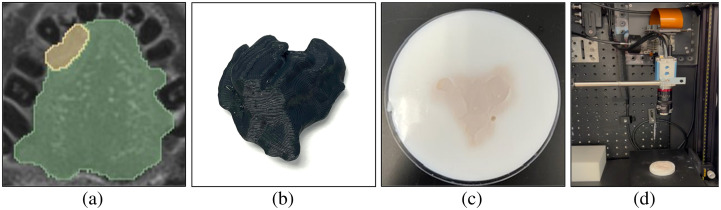
Optical phantoms of patient-derived tongue tumors as test data. (a) Contoured MRI of the tongue tumor. (b) 3D print of the tumor. (c) Optical agar phantom of the tumor. (d) SFDI system imaging phantom.

Optical phantoms were used to assess the performance of the DL model when experimentally collected images were passed to a model trained with *in silico* images. Figure 6 illustrates each stage within the phantom process. The contoured meshes used for *in silico* validation were 3D printed to create imprints for agar phantoms. These phantoms required two solutions: (i) non-fluorescent agar solution to represent the background tissue and (ii) fluorescent agar solution to represent the tumor. In both the background and tumor agar, India ink ($\mu_{a,\text{Background}}=0.0047$ and $\mu_{a,\text{Tumor}}=0.0093\;{\mathrm{mm}}^{-1}$) was the absorbing agent, and intralipid ($\mu_{s,\text{Background}}^{\prime }=1.44$ and $\mu_{s,\text{Tumor}}^{\prime }=1.42\;{\mathrm{mm}}^{-1}$) was the scatterer. The fluorescent agar solution included PpIX (5  μg/mL) as the fluorophore and iohexol as a contrast agent for computed tomography (CT) scanning. The background solution was poured into a dish, and the 3D-printed tumors were held at the surface of the solution to create a negative mold. Once the background agar solidified, the 3D-printed tumor was removed, leaving behind a negative imprint for the fluorescent tumor solution. The fluorescent solution was poured into the negative imprint to create a fluorescent tumor.

To model the true geometry, cone beam CT scans (Cios Spin, Siemens Healthineers, Erlangen, Germany) of the phantoms were taken to get the geometry and orientation of the tumor to the background agar. The CT scans were contoured in the 3D-Slicer software,^38^ to get a 3D mesh that was manually rotated to align with the images taken by the SFDI system. The calibrated mesh from the CT was then passed to our tissue simulator to obtain the ground truth for depth and concentration maps.

The training data for deep learning assumed the background healthy tissue was uniform and spanned the entire $50\text{-}{\mathrm{mm}}^{2}$ imaging field of view. To accommodate this, the background of the phantoms was designed to exceed the field of view. Although the images were originally the correct field of view, subsequent manual cropping was required to remove artifacts in the periphery of the images due non-uniform fluorescence filtering. The SFDI images were collected at a resolution of 10  pixels/mm and had varying dimensions based on the manual crop. If the manual crop created a region smaller than the required $50\text{-}{\mathrm{mm}}^{2}$ field of view, images required padding. To resize the image to have at least a $50\text{-}{\mathrm{mm}}^{2}$ field of view, the images were padded with the values within one standard deviation of the mean background intensity of non-fluorescent agar. As the pixel size at the imaging plane was not an even multiple of the pixel size of the training data (101×101 images at $0.5\;{\mathrm{mm}}^{2}$), an interpolation step was required to convert camera images. Specifically, to obtain images of 101×101  pixels at a resolution of 0.5  mm/pixel, the padded images were cropped to 505×505  pixels at the original camera resolution (0.1  mm/pixel), and box interpolation was used to reduce the resolution to 0.5  mm/pixel. This process was repeated for all reflectance, fluorescence, absorption, and scattering images. Processed images were automatically uploaded to an AWS S3 bucket for DL depth and concentration predictions.

## 3 Results

### 3.1 *In Silico* Results

**Fig. 7:**
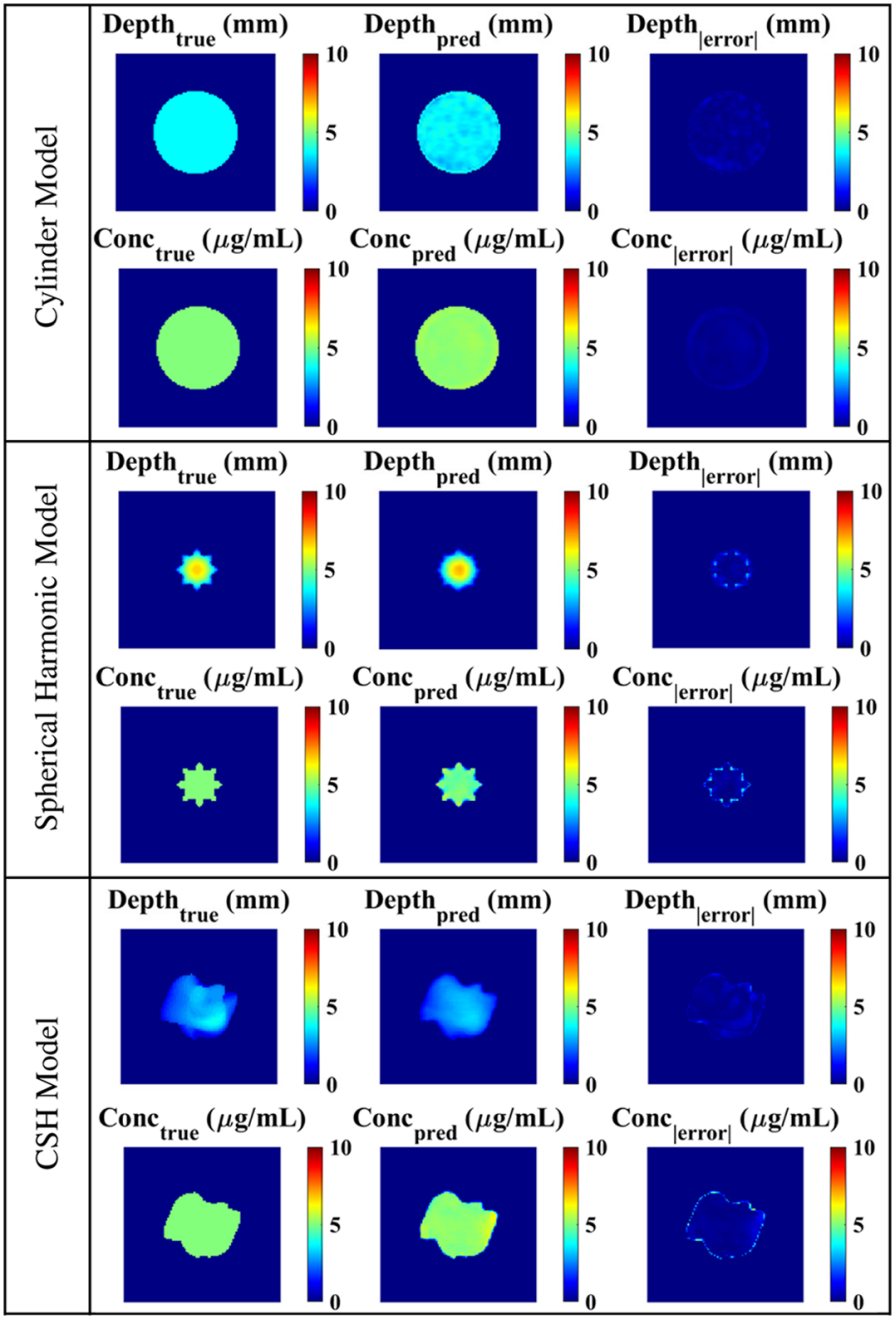
Architectures trained with different data types were tested with *in silico* data of the same class as the training data: the cylinder model was validated with 100 cylinders, the spherical harmonic model was validated with 100 spherical harmonics, and the CSH model was validated with 100 CSH.

To confirm that the architecture and parameters of the DL model were suitable, the models were tested with 100 images derived from the same data type as the training data: the cylinder model was tested with cylinders, the spherical harmonic model was tested with spherical harmonics, and the CSH model was tested with CSH. As shown in Fig. 7, each model made reasonable predictions for both depth and concentration. The cylinder model had an average (standard deviation) depth error of 0.36 (0.55) mm and a concentration error of 0.091 (0.12)  μg/mL. The spherical harmonic model had depth errors of 0.72 (1.04) mm and concentration errors of 0.18 (0.47)  μg/mL. The CSH model had the highest errors with an average depth error of 0.90 (1.28) mm and an average concentration error of 0.29 (0.59)  μg/mL.

**Fig. 8:**
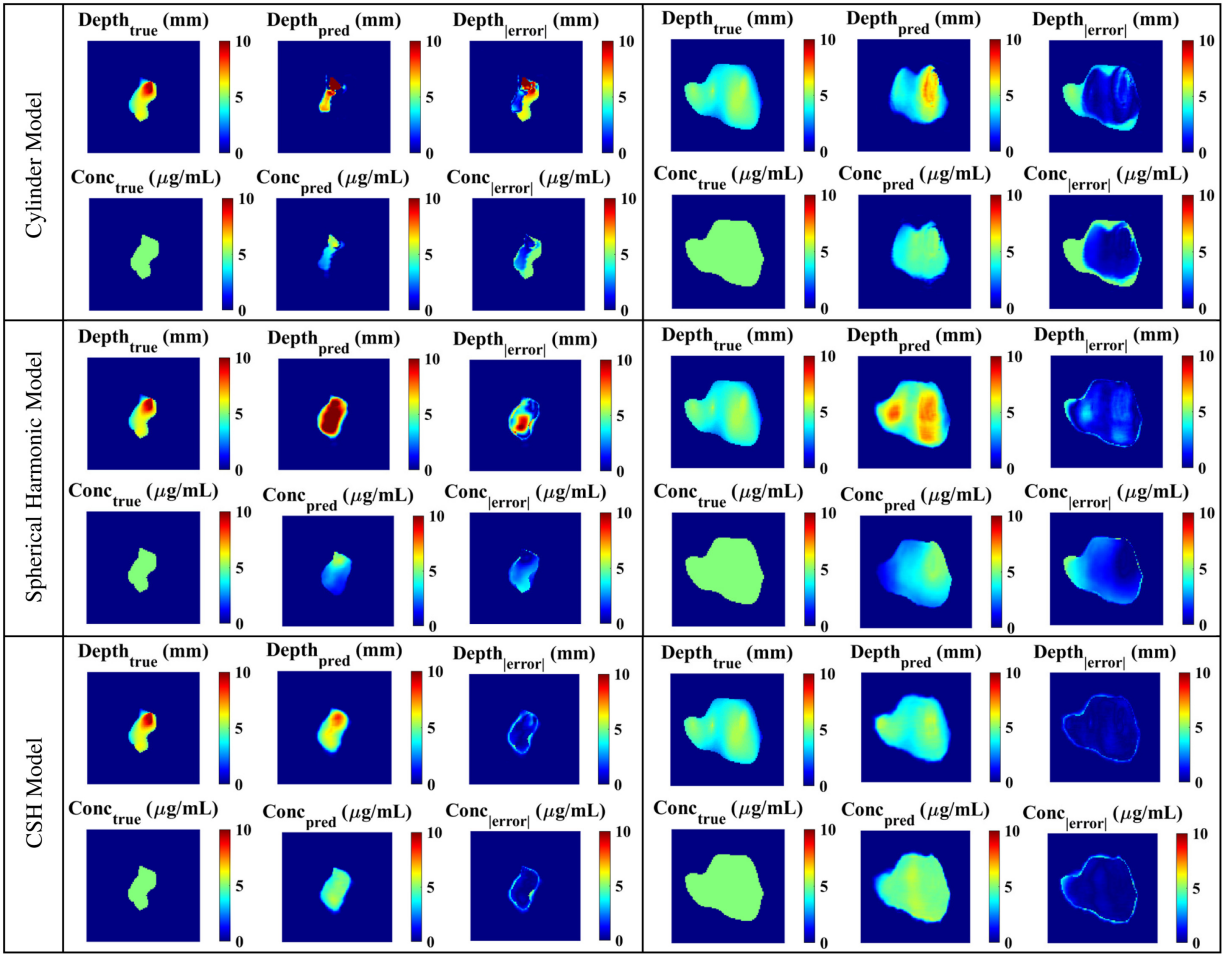
*In silico* images of patient-derived tumors for validating all models. As the complexity of the training data increased, the depth and concentration results improved for both unscaled (left column) and augmented (right column) tumors.

*In silico* SFDI fluorescence images and optical properties of the original and augmented patient-derived tongue tumors were passed to all three models. Although the architecture and parameters remained constant across all models, the model predictions differed significantly depending on the training data. Figure 8 illustrates DL predictions across models for a sample from the unscaled *in silico* set and a sample from the augmented set. Table 1 includes the predictive errors associated with each model when validated with the original patient-derived data (n=10). The model trained with cylinders had the overall highest error when testing with patient-derived tumor meshes. Complex geometric features within the testing set were not captured in the cylinder training set and the model could not accurately make predictions as shown in Fig. 8. The depth and concentration predictions had significant errors. The spherical harmonic model had significantly lower errors in concentration predictions (concentration $P\text{-value}=1.2\times 10^{-3}$) but did not demonstrate a significant improvement in depth predictions (depth P-value=0.34) when compared with the cylinder model. Due to the lack of asymmetry in this training set, many of the predictions had features intrinsic to spherical harmonics: deepest in the center with a gradual radial decrease in depth. The model trained with CSH had significantly lower errors than the cylinder model (depth $P\text{-value}=4.7\times 10^{-5}$ and concentration $P\text{-value}=5.4\times 10^{-10}$) and spherical harmonic model (depth $P\text{-value}=5.5\times 10^{-3}$ and concentration $P\text{-value}=1.6\times 10^{-4}$). As the complexity of the shape model increased and the data became more diverse, the model could learn geometric features intrinsic to real tumors.

**Table 1:** DL models were validated with the original unscaled and augmented patient-derived tumors. This table includes the depth and concentration average (standard deviation) errors associated with each DL model.

	Original tumors (n=10)		Augmented tumors (n=10)	
	Depth error (mm)	Concentration error (μg/mL)	Depth error (mm)	Concentration error (μg/mL)
Cylinders	4.2 ± 4.9	2.8 ± 2.1	1.62 ± 2.5	1.9 ± 2.0
Spherical harmonics	3.3 ± 3.6	1.78 ± 1.3	1.2 ± 2.0	0.9 ± 1.1
CSH	1.0 ± 1.5	0.5 ± 0.8	0.4 ± 0.6	0.4 ± 0.6

The improvement in DL predictions as training data complexity increased was apparent when the models were validated with the augmented patient-derived data (n=100). The cylinder model had significantly higher errors than both the spherical harmonic model (depth $P\text{-value}=2.6\times 10^{-3}$ and concentration $P\text{-value}=2.1\times 10^{-25}$) and the CSH model (depth $P\text{-value}=9.3\times 10^{-19}$ and concentration $P\text{-value}=5.7\times 10^{-63}$). The CSH model continued to have significantly lower error than the spherical harmonic model (depth $P\text{-value}=3.7\times 10^{-8}$ and concentration $P\text{-value}=9.6\times 10^{-15}$), indicating that the CSH had superior predictive performance when working with patient-derived data. The difference in errors between the two validation sets was likely due to the average depth of the data. As the average depths of the augmented tumors matched the average depths of the training set, the errors were consistently lower in the augmented validation set as shown in Table 1.

The unscaled and augmented tumor sets were also used to assess the effects of adding background fluorescence. The CSH model trained with background fluorescence was validated with *in silico* images of the unscaled and augmented test sets containing background fluorescence. The respective depth and concentration error for the unscaled set was 1.0±1.5  mm and 0.6±1.0  μg/mL, and the augmented set was 0.4±0.7  mm and 0.3±0.6  μg/mL. In comparison with the CSH model trained and tested with the same tumor sets without background fluorescence, there was no significant difference between depth and concentration estimates for the unscaled (depth P-value=0.9, concentration P-value=0.2) and augmented (depth P-value=1.0, concentration P-value=0.2) tumor sets.

**Fig. 9:**
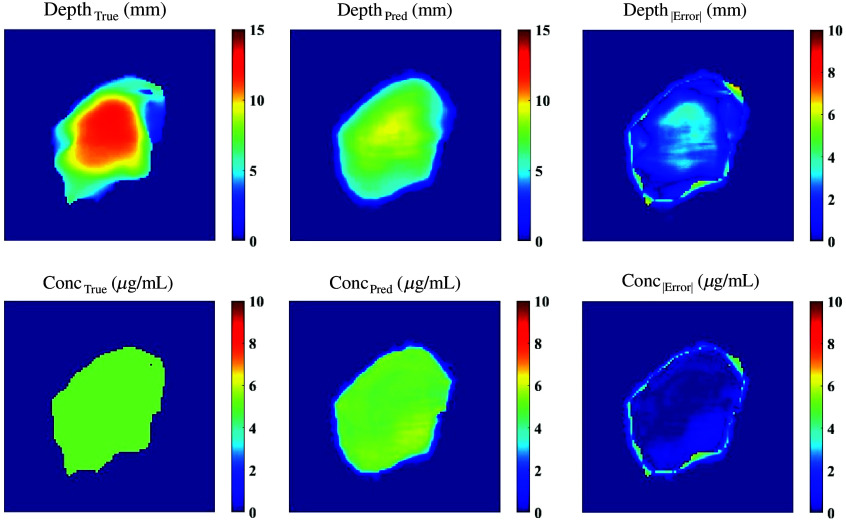
CSH model predictions for an *in silico* patient-derived tumor with a maximum depth of 12.5 mm. The average depth error is 1.83, mm and the concentration error is 0.32  μg/mL.

Figure 9 illustrates a patient-derived case with maximum depth (12.5 mm) that exceeds the ranges of depths (1 to 10 mm) included in the CSH training model. This case was specifically selected to assess what happens when the DL model encounters “out-of-range” depths (i.e., is there evidence of “AI hallucinations”?), with additional cases required in the future to assess this fully. Here, the depth predictions truncate at ∼10  mm and the error increases as depth increases.

### 3.2 Phantom Results

**Fig. 10:**
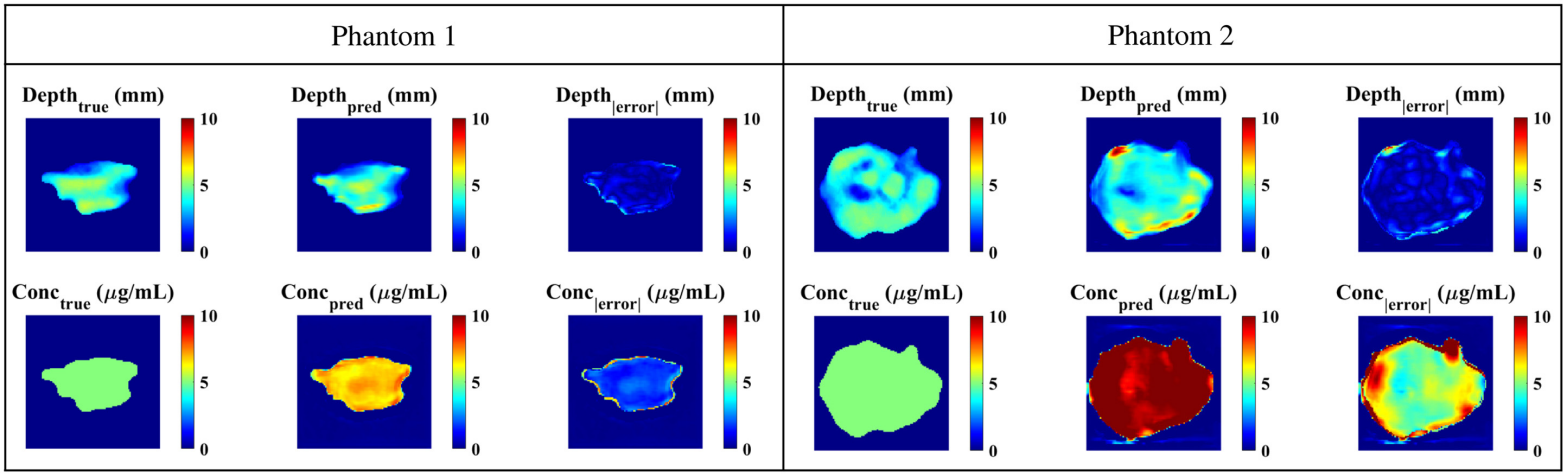
Phantom results from the CSH model trained with background fluorescence.

As the CSH model had the lowest errors *in silico*, it was used as the primary model for phantom studies. Figure 10 illustrates the predictions from this model on two patient-derived phantoms (maximum depths of 5.70 and 5.25 mm) made from separate batches of agar-based material. Phantoms 1 and 2 had average depth errors of 0.62 and 0.75 mm, respectively, and average concentration errors of 1.84 and 5.65  μg/mL. One possible source of error in the concentration map is the choice of PpIX fluorescence quantum efficiency used to train the DL model; here, we used η=0.046 for PpIX,^36^ but a number of values are reported in the literature.^39^ Batch-to-batch variability in phantom preparation may also contribute to concentration estimate uncertainties.

## 4 Discussion and Conclusion

This pre-clinical study demonstrates the potential to use CSH as an *in silico* oral cancer tumor model to train a DL-enabled SFDI system for fluorescence depth quantification. Simulation studies using 10 patient tongue tumor contours were used for initial validation, as well as to generate an augmented data set of 100 simulated cases for more comprehensive testing. Although initially focused on oral cancer, this work also establishes a generalizable deep learning architecture for SFDI-based fluorescence depth quantification to support future developments for other surgical oncology applications (e.g., breast, brain, and ovary).

Without a large database containing 3D meshes of patient-derived oral tumors for deep learning training, CSHs serve as a promising alternative. Training with patient-derived tumors would be optimal; however, contouring MRI images of oral cancer is time-consuming and prone to errors. In contrast, automatically generating synthetic tumor shapes offers a fast method of creating a diverse training set. Cylinders and spherical harmonics as tumor models were vital in the preliminary stages of developing a shape model as they served as a proof-of-concept for initial studies and led to the development of CSH as tumor surrogates. Although the primary concern associated with such synthetic datasets is that they are unable to capture the complexities of real data, the CSH shape model demonstrated its ability to capture geometric features intrinsic to patient-derived oral tumors in an initial test of 10 subjects.

Although the geometry of patient-derived tumors is captured by the training data, there are multiple limitations associated with this initial study, beyond the limited number of test samples. First, although the CSH model demonstrates its ability to capture the bulk geometries of real tumors, the shape model does not account for the complex infiltrative boundaries of oral tumors. Although in practice oral tumors are also defined on the bulk geometry, large margins are required to account for the infiltrative tumor boundaries. Pathology reveals that these boundaries contain irregular “finger-like” protrusions, satellite tumor nodules, and tumor buds.^40^ As diffuse optical imaging techniques have a limited ability to capture microscopic features, surgical margins will still be required. Future work should involve integrating small, localized regions of tumor to the shape model to mimic the infiltrative boundaries of real tumors. This addition is also required to assess our system’s limits in terms of fluorophore accumulation and diffuse optical imaging in small nodules. Second, the light propagation model assumed a homogeneous distribution of the fluorophore completely localized to the tumor. This simplified the calculations required for generating the fluorescence images and optical property maps; however, this assumption does not align with *in vivo* studies as tumors tend to have heterogeneous distributions of the fluorophore. Third, to simplify the training data, we assumed that the tumors were completely flush with the background and that the tissue surface was level. This approximation will not hold for real patient cases as tumors and healthy tissue have some degree of topography. To address this, phase-shifted profilometry will be integrated into the SFDI workflow to correct for irregular surfaces,^41^ in combination with Monte Carlo simulations of light propagation.^32^ Further assessment of the optical properties and reproducibility of the phantom recipe is required. Finally, only two phantoms were included here as an initial proof-of-concept demonstration, but future work will include results from additional patient cases and investigate other fluorophores of known quantum efficiencies.

One limitation of our approach is the assumption of a known fluorophore quantum efficiency (η) to relate fluorescence yield ($\eta \mu_{a,f}$) to concentration (μg/mL). Although this is feasible in a controlled laboratory setting (e.g., PpIX in a known solvent), in the *in vivo* setting, it is known that PpIX η depends on unknown local environmental factors (e.g., pH, oxygen, and temperature).^39^ In this case, it may only be possible to recover PpIX concentration estimates within a linear scale factor of the known concentration, as previous authors have suggested for PpIX fluorescence reconstruction.^36^ Future studies will be required to assess how quantum efficiency variations affect depth and concentration estimates under more realistic conditions, not only for PpIX but potentially for other NIR fluorophores that may exhibit different *in vivo* behavior.

The exact depth at which DL estimates start to degrade due to the limited penetration of near-infrared light is subject to more extensive evaluation. The simulation and phantom test cases reported here were specific to nominal absorption coefficients ($\mu_{a}<0.01\;{\mathrm{mm}}^{-1}$ at 632 nm), showing <0.4-mm error for tumors with depth <1  cm. Additional testing is required across a broader range of conditions, including cases with higher absorption coefficients. Certainly, accurate depth estimation will not be feasible in reflectance mode for all oral cancers, particularly late-stage cases with depths in the centimeters (e.g., T3 and T4).^42^ Nonetheless, although this paper focused on depth quantification suitable for *in vivo* imaging of mucosal oral cancer tumors (i.e., “iceberg scenario” of depth to bottom tumor surface), in the future, this system will be applied to quantify margin thickness of “inverted” tumors (i.e., “submarine scenario” of depth to top tumor surface), in which depth quantification is required down to a 5-mm depth to confirm margin status.^11^ For both *in vivo* and *ex vivo* scenarios, future pre-clinical experiments in animal models with complex, heterogeneous optical properties and fluorophore distributions will be essential to validate this technology under more realistic clinical conditions.

## Data Availability

Available upon reasonable request to the corresponding author.
